# Temporal change in functional rarity in marine fish assemblages

**DOI:** 10.1098/rspb.2022.2273

**Published:** 2023-02-22

**Authors:** Faye Moyes, Isaac Trindade-Santos, Anne E. Magurran

**Affiliations:** ^1^ Centre for Biological Diversity, School of Biology, University of St Andrews, St Andrews KY16 9TH, UK; ^2^ Marine Macroevolution Unit, Okinawa Institute of Science and Technology Graduate University, 1919-1, Tancha, Onna-son, Kunigamigun, 904-0495, Okinawa, Japan

**Keywords:** marine fish, functional rarity, structural change, biodiversity, species abundance distribution, trait abundance distribution

## Abstract

Recent research has uncovered rapid compositional and structural reorganization of ecological assemblages, with these changes particularly evident in marine ecosystems. However, the extent to which these ongoing changes in taxonomic diversity are a proxy for change in functional diversity is not well understood. Here we focus on trends in rarity to ask how taxonomic rarity and functional rarity covary over time. Our analysis, drawing on 30 years of scientific trawl data, reveals that the direction of temporal shifts in taxonomic rarity in two Scottish marine ecosystems is consistent with a null model of change in assemblage size (i.e. change in numbers of species and/or individuals). In both cases, however, functional rarity increases, as assemblages become larger, rather than showing the expected decrease. These results underline the importance of measuring both taxonomic and functional dimensions of diversity when assessing and interpreting biodiversity change.

## 1. Introduction

Contemporary ecological communities are experiencing biodiversity change that has little precedence in the historical record, with marine systems among those in which this change is particularly marked [1–3]. This biodiversity crisis underlines the importance of measuring biodiversity in robust and ecologically meaningful ways. But because biodiversity is a multifaceted concept [4,5], it also raises questions about the extent to which information on change in one dimension of diversity, such as taxonomic diversity, sheds light on change in other dimensions, such as functional diversity. Growing evidence that ecosystems are being restructured along multiple dimensions of biodiversity [6] underlines the need for improved understanding of the linkages between these dimensions.

Ecological assemblages typically consist of a few common and many rare species, a pattern that is described by a species abundance distribution (SAD). Species that are considered to be taxonomically rare occupy the lowest ranks in this distribution [7,8], with other categorizations of rarity drawing on species occurrence data, and/or features such as habitat specificity (e.g. [9]). A recent macroecological analysis [10] showed that increases in taxonomic rarity are widespread. Such shifts have been attributed to immigration and warming [11–16], and may occur alongside an increase in assemblage size due to greater number of species and/or individuals. Taxonomically rare species could contribute unusual trait combinations to a system [17,18] and play an important role in supporting ecosystem functioning [19,20]. Temporal change in taxonomic rarity thus has the potential to shed light on underpinning changes in functional rarity. However, the biodiversity literature contains many examples of cases where change in one attribute of diversity is uncorrelated or only weakly correlated with another (e.g. [21,22]). Moreover, a taxonomically rare species can have a dominant trait value and vice versa. Therefore, even though metrics of functional rarity can be weighted by taxonomic abundance [23], it does not necessarily follow that trends in taxonomic rarity, and trends in trait (functional) rarity will coincide. To predict whether change in taxonomic rarity and change in functional rarity are correlated, we need to understand how metric responses are shaped by shifts in the underlying SAD.

If an assemblage gains more biomass, or larger numbers of individuals, the number of species in the assemblage is expected to rise, but in a nonlinear way. This is the principle that underlies rarefaction analyses used to make fair comparisons between assemblages [4,24,25]. Owing to the constraints imposed by the uneven distribution of species that characterize SADs [26] other assemblage properties will also change as an assemblage grows (or shrinks). For example, larger assemblages are generally less (taxonomically) even than smaller ones [27]. We therefore expect larger assemblages to exhibit increases in taxonomic rarity. However, trait abundance distributions (TADs) tend to be more even than SADs (e.g. [28]). This higher evenness in TADs [29] suggests that functional rarity may be less responsive to a change in assemblage size than taxonomic rarity. To explore these questions, we construct a null model taking account of both observed species and TADs and in which individuals are progressively drawn at random from a gamma [30] assemblage to construct local assemblages of different size. Departures from this null will shed light on how rare trait combinations are conserved or lost, as assemblages change in size.

**Figure 1.**
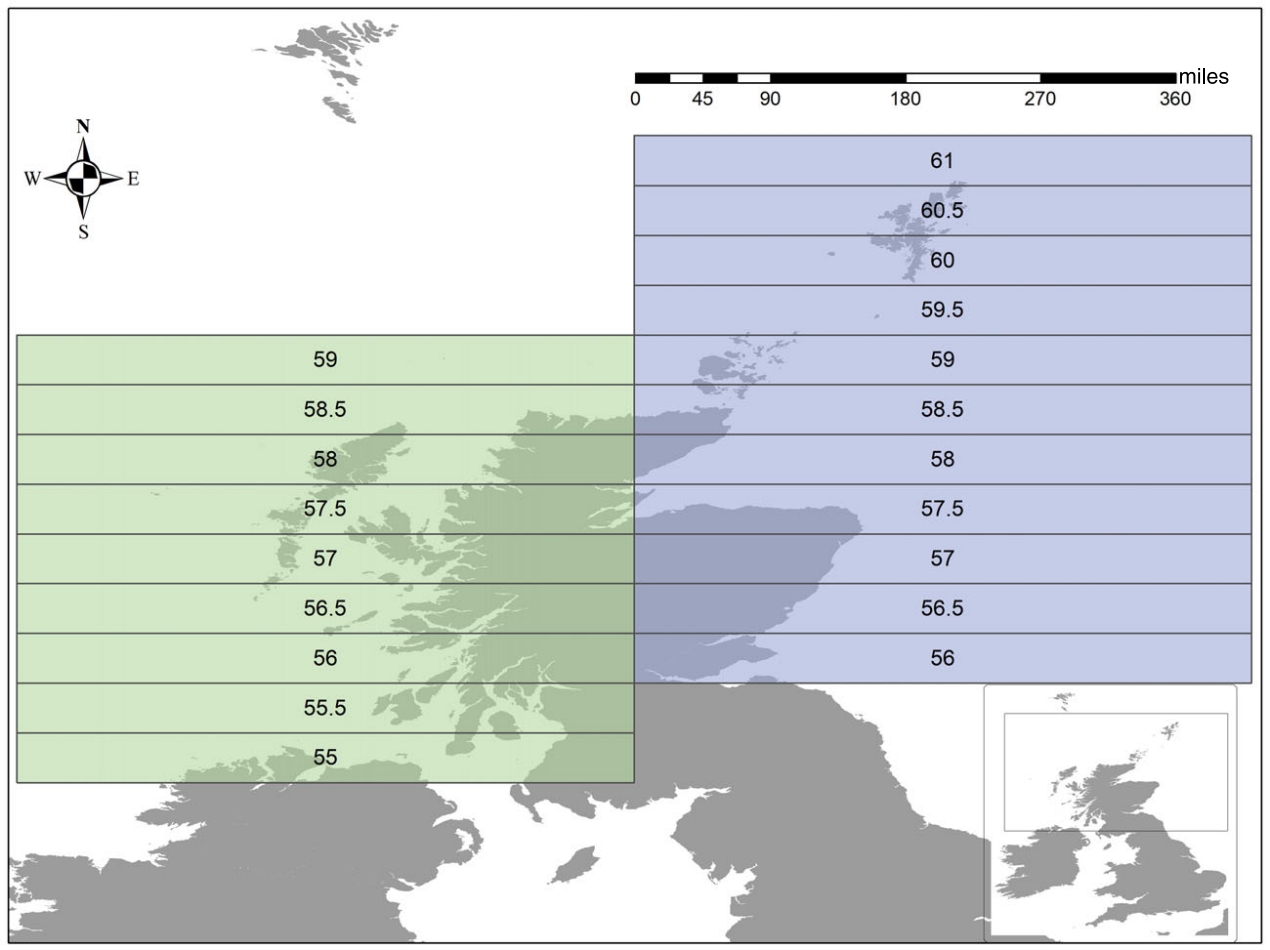
Map showing the latitudinal bands with the central latitude added in text. The west coast system is shaded in green with the east coast in blue (this colour scheme is consistent throughout the paper).

Here we focus on two Scottish marine fish assemblages (figure 1), each sampled by scientific trawling over a period of three decades. Although matched by latitude, these assemblages belong to different marine ecosystems: the seas to the west of Scotland are part of the Celtic-Biscay Shelf ecosystem [31] while those to the east of Scotland are placed in the North Sea ecosystem [32]. These systems share many, but not all, fish species, but have different dominant species, and differ in how species dominance changes over time [33]. They thus provide an interesting test case in which to ask whether shifts in taxonomic rarity are a proxy for change in functional rarity as well as whether these biodiversity changes are consistent across different geographical regions.

## 2. Methods

### (a) Study location

The data used in this work were sourced from the International Council for the Exploration of the Sea (ICES) and are taken from two standardized scientific trawl surveys incorporating the ICES areas VIa (west coast system), IVa and IVb (North Sea) [31,32] (east coast system). Each species record contains a precise geographical location and numerical species abundance represented by CPUE (catch per unit effort), which in this instance refers to the number of individuals of a given species caught per hour using a tow duration of half an hour. Here we use ICES rectangles to form the boundaries of assemblages. These ICES rectangles are freely available for download on the ICES website [34] and represent 30′ latitude by 1° longitude in a grid cell. In this work, we chose latitudinal bands which were created by merging the ICES rectangles longitudinally. This produced 11 ‘bands’ on the east and 9 on west coast (see map in figure 1; for further information see electronic supplementary materials).

The west coast system (which is part of the overall Celtic Seas ecoregion) is mostly relatively shallow, particularly in the Irish Sea area where depth is typically less than 100 m, and comprises a variety of habitats [35], including rocky outcrops and sandbanks. The system is largely sheltered from the winds and currents of the North Atlantic and water circulation patterns are influenced by freshwater discharge from rivers such as the Severn and the Shannon as well as the many sea lochs found on the west coast of Scotland [36]. The North Sea system is semi-enclosed and includes the Norwegian Deeps in the north-eastern portion where depths can be up to 700 m. Much of the remainder of the ecosystem is relatively shallow with large estuarine areas. The habitats here are largely dominated by sand, mud and some harder grounds around the Orkney and Shetland islands.

### (b) Data handling and analysis

Each latitudinal band produced a 30-year time series. We employed sample-based rarefaction to reduce bias due to sampling effort [8,24] (for further details on the process, see electronic supplementary materials). To do this, we resampled the data based on the minimum number of samples where a sample consisted of all trawl information for a single year within a single latitudinal band. We also filtered records to ensure that no crustaceans or other non-finfish were included in our final dataset. This process left us with 116 species in the west coast pool and 121 species in the east coast pool.

We first computed numerical abundance (N) and species richness (S) at each time step within each latitudinal band to understand how assemblage size is changing. To quantify temporal change in the metrics, we fitted an ordinary least squares (OLS) linear regression model [37]. We also computed the median absolute deviation (MAD), using the mad function from the stats package in base R [38], to examine the variation in trends within each system.

Next, we assembled information on the fish diversity of each of these latitudinal bands at each time step. To calculate taxonomic rarity, we used the ‘funrar’ package in R [39]. The taxonomic rarity of a species within an assemblage is measured by using the inverse of relative abundance. Very rare species have a value close to 1 while common/abundant species will approach 0 (for equation, see electronic supplementary materials). This score is assigned to each species, and therefore, to produce an assemblage level measure, we used the mean score for the species present in a given year. We also calculated Simpson's evenness (taxonomic evenness) [8] and species richness (S) in each case.

To compute functional rarity, we selected 11 traits, both continuous and categorical, reflecting the ecological roles of the species. Traits were sourced using the most recent release of FishBase [40] (for further details on trait choice, see electronic supplementary material, table S1 and trait choice). To understand better the shape of these trait distributions we examined the kurtosis and skewness of the continuous traits (see electronic supplementary material, table S3). This was done using the moments [41] package in R. We then generated the multidimensional functional space, based on Gower distance, occupied by each assemblage using the R function (*quality_funct_space()*) developed by Maire *et al*. [42]. Functional rarity as used here is weighted by abundance and corresponds to the mean pairwise distance between species within the assemblage (see electronic supplementary material, figure S1 for a pictorial representation). For any species, distance is measured between it and all others in the assemblage, with its functional rarity computed as the mean of these distances (see electronic supplementary materials for equation). As with taxonomic rarity, functional rarity ranges between 0 and 1, with rarer species tending towards 1 and more common species tending towards 0 (for equation see electronic supplementary materials). Additionally, as with taxonomic rarity, this is a species-level metric and we therefore used the mean rarity values of those species present at each time step within an assemblage [23,43].

Given the potential importance of the distribution of trait values in shaping the response of functional rarity to shifts in assemblage size [28,29], we also calculated the functional evenness of the trait distribution, and the skewness and kurtosis (calculated using the moments package in R [41]) of the species-level functional rarity values within each latitudinal band at each time step. Finally, to understand whether functionally rare species recruited to these assemblages are also taxonomically rare, we re-computed the functional rarity metric with no abundance weighting (figure 4*g,h*).

### (c) Null model

Separate null models (see electronic supplementary material, figure S3) were constructed for the two coastal assemblages. First, a subset of species (58 for the west coast and 55 for the east—these are the typical maximum numbers observed in a latitudinal band) was selected at random from the overall species pool of a given coastal system. A data frame of trait values for these species was created. Next, we re-assigned trait values to each species in this null gamma assemblage by independently randomizing the vector of each trait in the data frame. This shuffling broke any inherent correlation between traits and produced a null gamma assemblage in which each of the species had a randomly allocated set of trait values. Species retained their relative numerical abundance, as expressed in the original dataset. Following this, *n* = 100 individuals were sampled, at random, from the null gamma assemblage. The same assemblage properties as before, namely total number of species (S), mean functional rarity, skewness and kurtosis of functional rarity, functional evenness, mean taxonomic rarity and Simpson's evenness, were computed after each draw. Next, the value of *n* was increased in progressive steps (this step was a proportion of the total n in the chosen assemblage and ranged between 50 and 2000), with assemblage properties again computed at each step, until maximum *n* in the subset is reached. The trait array was then re-shuffled before the whole model was re-run. This was repeated 1000 times, with the mean and standard error (95%) of each assemblage property computed on each run. In all cases, we constructed a S(N) rarefaction plot as a check that the null model was behaving as expected (see electronic supplementary material, figure S4). The whole procedure was then repeated five times, starting with a new draw of either 55 or 58 species from the regional assemblage.

The final output of the model produced a data frame of metrics at each value of N. We used this to visualize the relationship between metrics in the null. The model performed consistently using a range of initial sample pools (see electronic supplementary material, figure S5), thus providing evidence of the robustness of our results.

## 3. Results

**Figure 2.**
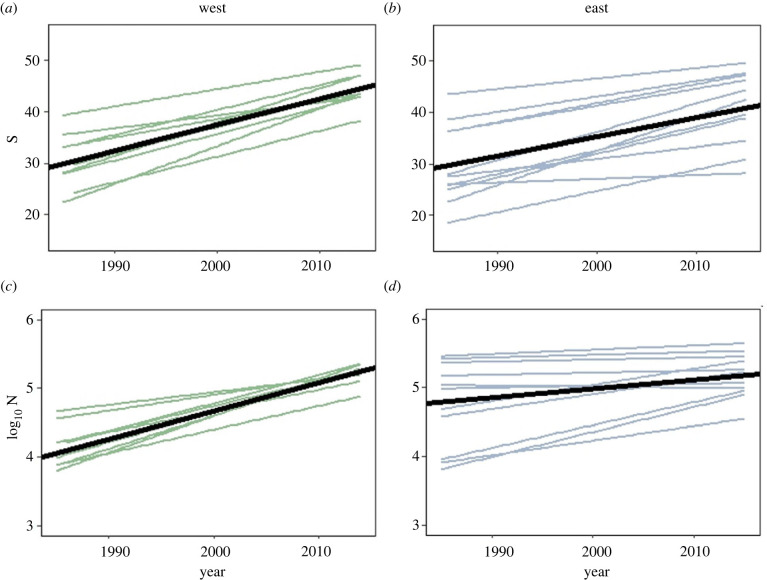
Slopes of change for each metric by coastal system (the west coast is shown on the left and coloured in green, with the east coast on the right and coloured blue). The darker line is the common trend (computed as the OLS regression of all the time series) and the lighter lines the individual latitudinal bands. The figure shows the greater heterogeneity of slopes on the east coast as compared to the west (see text for details). The overall slopes (solid black lines) are significant for both metrics in both systems. East: S *p*-value < 0.0001, N *p*-value = 0.0004; west: S *p*-value < 0.0001, N *p*-value < 0.0001.

Our analyses revealed that both coastal systems increased in assemblage size (S and N) over the duration of the time series (figure 2). In addition, we observed greater variability in trends in the east coast than the west for all metrics apart from evenness. Results of the MAD were as follows. West coast: S = 0.185 and N = 1863. East coast: S = 0.206 and N = 3375.

**Figure 3.**
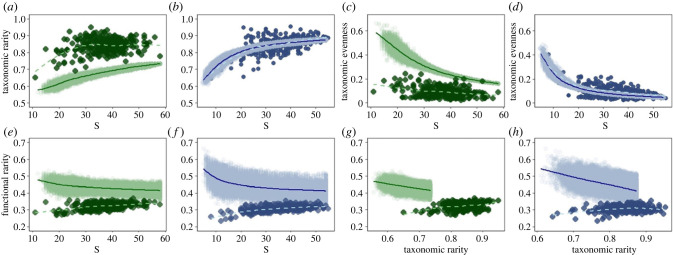
Relationship between null and observed. Observed values (yearly results by latitudinal band) are shown as solid points, with the distribution of null results indicated by the shaded area. In both cases trends are indicated using a loess fit, computed using the stat_smooth(method = ‘loess’) option in ggplot2. West coast results are shaded green and east coast blue, with the observed loess as a pale dashed line and the null as a darker solid line.

Our null model showed, as expected, that as the number of individuals in an assemblage increases, so too does the number of species, but in line with expectation on a saturating curve (see electronic supplementary material, figure S4). As assemblage size increases, taxonomic rarity is expected to increase, and evenness to decline, and this is what we found (figure 3*a–d*). On the east coast levels of taxonomic rarity and evenness were aligned with the null while west coast assemblages had greater taxonomic rarity and less evenness than expected.

In addition, the null model predicted that functional rarity should decline as assemblages grow in size, and as taxonomic rarity increases (figure 3*e–h*). However, in neither the west coast system nor the east coast system (figure 3) did the observed data show these trends. In both systems, functional rarity showed a weak increase in response to both richness and taxonomic rarity and occurred at lower levels than predicted (figure 3*e–h*). The same patterns were evident when the null model was re-run with different gamma assemblages indicating that the results are robust against variation in initial composition (see electronic supplementary material, figure S5).

**Figure 4.**
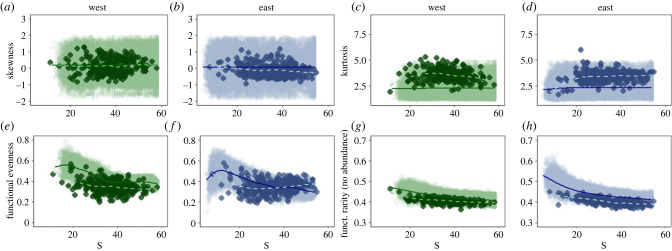
Relationship between null and observed. As with figure 3, observed values are shown as solid points, with the distribution of null results indicated by the shaded area and trends shown using a loess fit. The west coast results are shaded green and east coast blue, with the observed loess as a pale dashed line and the null as a darker solid line.

In both west and east systems, values for the skewness of the observed distributions of functional rarity (figure 4*a,b*), plotted in relation to S, were nested within the null, with average trends close to zero in both cases. There was also overlap between observed and null levels of functional evenness (figure 4*e,f*) and in the kurtosis of functional rarity (figure 4*c,d*). However, in this latter case the distributions of observed functional rarity were moderately leptokurtic (median overall kurtosis: west null = 2.08, observed = 3.5; east null = 2.12, observed = 3.45; see electronic supplementary material, figure S6). Finally, when functional rarity was re-computed ignoring both the species abundance and TADs, the trends in both observed and null were closely matched (figure 4*g,h*).

## 4. Discussion

Assemblages on both coasts are increasing in richness and in numerical abundance. These shifts in assemblage size should lead to increases in taxonomic rarity and decreases in (taxonomic) evenness, and this is what we found. This indicates that the directionality of changes in these taxonomic properties of these species' abundance distributions is consistent with our expectation based on a random draw from the gamma assemblage, albeit with some differences in the magnitude of the response between coasts. All other things being equal, as the null shows, we also expected this observed increase in assemblage size to lead to a decrease in functional rarity [29]. However, we found the opposite with both systems exhibiting a weak positive increase in functional rarity, as they gained more species. Moreover, and in further disagreement with the null, observed functional rarity was broadly maintained as taxonomic rarity increased.

A species’ functional rarity value is dependent not only on its own trait combination and abundance but also on the trait combinations and abundance of all other fish in the assemblage [23,39,44]. The shape of this TAD, for example, its degree of skewness and kurtosis, will determine not just the level of functional rarity, but also shed light on the processes involved in community assembly [28,29]. Our analysis, which took account of the TAD as well as the SAD, detected no disagreement between the observed and null for trends in relation to increases in assemblage size for either functional evenness or the skewness of the functional rarity distribution. On the other hand, distributions of observed functional rarity tended towards leptokurtosis which could help explain why our observed values of functional rarity are lower than the null expectation (figure 3*e,f*). This interpretation is supported by the analysis of functional rarity in which both SAD and TAD were ignored (figure 4*g,h*). Here we uncovered a decline in functional rarity in larger assemblages, as predicted by our initial null. We therefore conclude that the functionally rare species that are entering these local assemblages are less abundant than is predicted from a random draw from the gamma assemblage.

A striking feature of our results is that the observed relationships between functional rarity and richness, and between functional rarity and taxonomic rarity were generally weak with relatively little trend in the metric in response to shifts in assemblage properties. Functional evenness also changed little with assemblage size, particularly in the east coast system (figure 4*f*). Taken together these findings suggest that the functional properties of these marine fish assemblages are conserved as assemblage size changes. Working with within-trait variation, Gross *et al.* [28] reported more even abundance distributions of trait values within dryland plant assemblages than would be expected by chance. Such patterns help maximize local multifunctionality [19,28,29]. In our case, we did not find any marked discrepancy between null and observed functional evenness, but we computed functional evenness across eleven traits rather than within a single trait. It would be interesting examine the TAD at the individual as well as the species level, but we were unable to do this due the unavailability of intraspecific trait information.

Our analysis also uncovered interesting differences between the two systems. For example, we observed higher levels of taxonomic rarity relative to the null expectation (figure 3*a,b*), as well as reduced evenness, for given levels of richness, in the west coast system (figure 3*c*) as opposed to the east coast system (figure 3*d*). Since increased taxonomic rarity can be associated with habitat complexity [45–47], this result could reflect the increased structural heterogeneity of the west coast, as well as contrasts in warming trends, and/or recovery from historical fishing pressures [33,48]. Historically, the east coast system (North Sea region) has been more heavily exploited than the west coast system (Celtic Sea area), but, since the beginning of this time series in 1985, fishing pressure has been largely similar in both areas (see electronic supplementary material, figure S7). The differences in taxonomic diversity could also be linked to the increased variability in trends on the west coast (figure 2). Nonetheless, the observed relationship between functional rarity and richness, and the level of functional rarity, were similar in the two coastal systems suggesting that environmental filtering and niche differentiation could be important in shaping the distribution of traits in both cases [29].

To date, investigations of temporal change in marine ecosystems have focused on single populations [49,50]. However, it is becoming clear that multi-species, assemblage-based analyses [51,52], which include information on functional and taxonomic diversity, and potentially also phylogenetic diversity, will be important in tracking biodiversity change in these systems, predicting their resilience in the face of anthropogenic pressures [53], and in shaping conservation decisions and designing fisheries policy [54–56]. As our investigation has shown, different dimensions of biodiversity change are not necessarily correlated. Understanding how this complexity arises, and what its consequences are for the maintenance of ecosystem function, is a substantial future challenge. Null models, as employed here, represent a powerful means of elucidating the processes that underpin assemblage restructuring [56]. For example, it would be interesting to use a null model approach to examine the interactions between environmental filtering and shifts in assemblage size, particularly when the latter is a response to an increase in carrying capacity linked to climate change. Unravelling the mechanistic links between trends in functional and taxonomic diversity will also be important, in, for instance, discovering the extent to which functional rarity is linked to whether a species is a winner or loser during biodiversity change [33,57].

In conclusion, analyses of the two coastal systems revealed that trends in taxonomic rarity are an inadequate proxy for trends in functional rarity, and that the ongoing increases in assemblage size can have complex, and context-dependent, consequences for assemblage biodiversity. A clearer understanding of the potential drivers of change in functional rarity can assist with more targeted conservation plans and fisheries management, and it is already clear that shifts in community rarity have implications for ecosystem resilience [17,20,58,59]. Our study highlights the importance of taking an integrative and multidimensional approach to protect, maintain and sustain the functional integrity of marine fish assemblages [60–62].

## Data Availability

All data are publicly available via the DATRAS portal (http://datras.ices.dk/Data_products/Download/Download_Data_public.aspx). Code is included in the electronic supplementary material [63]. Additional information is also provided in the electronic supplementary material [63].
